# Shotgun metagenomic dataset of seawater bacterial communities from Pari Islands, Indonesia

**DOI:** 10.1128/mra.01476-25

**Published:** 2026-03-23

**Authors:** Buntora Pasaribu, Christopher Vincent Mishael Dilens, Muhammad Wahyudin Lewaru, Diah Ayuningrum, Mufti Petala Patria, Donny Juliandri Prihadi, Noir P. Purba, Mochamad Untung Kurnia Agung, Ismail Maqbul, Sulistiowati Sulistiowati

**Affiliations:** 1 Department of Marine Sciences, Faculty of Fisheries and Marine Sciences, Universitas Padjadjaran 61809, Bandung, Indonesia; 2 Shallow Coastal and Aquatic Research Forensic (SCARF) Laboratory, Faculty of Fishery and Marine Sciences, Universitas Padjadjaran, Bandung, Indonesia; 3 Marine Science Study Programme, Faculty of Fisheries and Marine Sciences, Universitas Padjadjaran 61809, Bandung, Indonesia; 4 Department of Aquatic Resource Management, Diponegoro University 95445, Semarang, Indonesia; 5 Department of Biology, University of Indonesia 64733, Depok, Indonesia; 6 Department of Fisheries, Faculty of Agriculture, Gadjah mada University 59166, Yogyakarta, Indonesia; Wellesley College, Wellesley, Massachusetts, USA

**Keywords:** metagenomics, microbial communities

## Abstract

Pari Island is located in Seribu Islands, Indonesia, and is well-known for its marine biodiversity. Shotgun metagenomic sequencing was performed using the DNaseq-G400 platform, and bioinformatics approaches were applied to analyze the sequence data.

## ANNOUNCEMENT

Pari Island is a region of significant ecological value with rich marine biodiversity, including *Ascidia* spp., coral reefs, mangroves, and seagrass (1–3). Furthermore, Pari Island currently faces a severe environmental crisis that is influenced by various tourist activities, climate change, and pollution (3–5). However, the data on microbial communities in the water column are still unknown.

Seawater samples were collected in August 2024 from Pari Islands (5.85205°S, 106.60571°E), with measured conditions of temperature (29.55°C), salinity (32 ppt), pH (7.25), and dissolved oxygen (6.06 mg/L). Two-liter water samples were aseptically filtered using a vacuum filter with a 0.22-μm nitrocellulose membrane (Sartorius, Germany). The filter membranes used for filtration were then employed in the subsequent process. DNA extraction was performed using the ZymoBIOMICS DNA Kit (Zymo Research, USA). DNA quality was assessed using agarose gel electrophoresis and the Qubit BR-DNA Assay (Thermo Fisher Scientific, USA), following the manufacturer’s instructions. DNA concentration was quantified using Qubit fluorometer dsDNA BR Assay (Thermo Fisher Scientific), and 13–15 ng/μL of each sample was used for sequencing, following the manufacturer’s instructions. The DNA library was prepared through end-repaired adapter and PCR amplification using the MGIEasy FS DNA Library Prep Kit (MGI Tech, China). Sequencing was carried out on MGI DNBSeq-G400 (MGI Tech) in the 2 × 150 bp paired-end mode, using the DNBSeq-G400RS High Throughput Sequencing Kit (FCL PE150) (MGI Tech). The raw data and quality of reads were obtained using the DNBSeq-G400RS, as detailed in Table 1 (6). Raw sequencing reads were evaluated for quality using fastp v1.0, and adapter sequences, along with low-quality bases, were trimmed from both ends to improve data reliability for downstream analyses (7, 8). The metagenomic assembly was performed using Metaspades v4.2.0 with the k-mer size set to 31 (9) (https://doi.org/10.5281/zenodo.18476699). The results of the metagenomic assembly were evaluated and compared with the closest reference using metaQUAST (10). Functional and taxonomic analyses were performed using Diamond v0.9.25 (11) + MEGAN v6 (12). Diamond v0.9.25 was used to align all DNA fragments to a protein database. The alignment results were then analyzed for taxonomic and functional information using MEGAN v6.

**TABLE 1:** Accession and data for metagenome information for the analyzed seawater samples

Sample	NCBI BioProject	SRA accession no.	No. of raw sequence reads	No. of reads that passed QC	N50 (bp)	Max contig
P1	PRJNA1218935	SRX27269221	40,073,216	40,073,120	360,742	121,199
P2	PRJNA1218910	SRX27269222	40,286,300	40,286,186	290,644	90,545

Taxonomic analysis revealed that Proteobacteria was the most abundant phylum (75.5%), followed by Cyanobacteria (14.5%), with minor contributions from viruses, eukaryotes, and other organisms (10%) (https://doi.org/10.5281/zenodo.18476606). The distribution of the most abundant bacterial species identified through metagenomic analysis demonstrated *Prochlorococcus marinus*, *Synechococcus* sp. *M16.1*, *Synechococcus* sp. *TAK9802*, *Methylobacterium oryzae*, *Candidatus Pelagibacter* sp. *FZCC0015*, *Candidatus Pelagibacter* sp. *RS39*, *Cognatishimia activa*, and *Thalassovita* sp. Metagenomic functional profiling revealed that metabolism (225,000 reads) was the predominant functional category in the microbial community of the water column sample, followed by energy production (75,000 reads) and protein processing (73,000 reads). Functional analysis of metabolism identified amino acid transport and metabolism (60,000 reads) as the most abundant metabolic function, followed by energy production and conversion (40,000 reads), inorganic ion transport and metabolism (38,000 reads), carbohydrate transport and metabolism (32,000 reads), lipid transport and metabolism (21,000 reads), and coenzyme transport and metabolism (20,000 reads).

## Data Availability

The sequences were deposited and are available in an NCBI SRA data set under BioProject numbers PRJNA1218935 and PRJNA1218910. The accession numbers are listed in Table 1.
